# Exploring Differential Patterns of Dissociation: Severity and Dimensions Across Diverse Trauma Experiences and/or Attention-Deficit/Hyperactivity Disorder Symptoms

**DOI:** 10.3390/bs15070850

**Published:** 2025-06-24

**Authors:** Rosario Esposito, Eduardo Maria Schettino, Veronica Buonincontri, Carmine Vitale, Gabriella Santangelo, Gianpaolo Maggi

**Affiliations:** 1 Department of Psychology, University of Campania “Luigi Vanvitelli”, 81100 Caserta, Italy; rosario.esposito@unicampania.it (R.E.); gabriella.santangelo@unicampania.it (G.S.); 2 School of Cognitive Psychotherapy (SPC), 80122 Naples, Italy; 3 Santa Maria del Pozzo Hospital, 80049 Somma Vesuviana, Italy; 4 Department of Medical, Motor and Wellbeing Sciences, University of Naples Parthenope, 80133 Naples, Italy; 5 ICS Maugeri Hermitage, 80145 Naples, Italy; 6 Department of Advanced Medical and Surgical Sciences, University of Campania “Luigi Vanvitelli”, 80138 Naples, Italy

**Keywords:** dissociation, Attention-Deficit/Hyperactivity Disorder (ADHD), trauma, Post-Traumatic Stress Disorder (PTSD), adverse childhood experiences, dissociative experience scale

## Abstract

Dissociative symptoms may result from both neurobiological conditions, such as Attention-Deficit/Hyperactivity Disorder (ADHD), and traumatic events/exposure, such as Post-Traumatic Stress Disorder (PTSD) and Adverse Childhood Experiences (ACEs). However, identifying whether dissociative manifestations are associated with ADHD symptoms or trauma-related manifestations may drive clinicians to select the most effective intervention. Four hundred participants from the general population completed an online survey and were classified based on the presence of PTSD, ACEs, or ADHD symptoms. We compared the severity of dissociation and its dimensions across groups using the Dissociative Experiences Scale-II (DES-II) and explored its association with ADHD symptoms, PTSD manifestations, and ACEs. Dissociative symptoms were more pronounced in individuals with combined ADHD and PTSD or ACEs, but a hierarchical pattern of dissociation severity was also observed in isolated symptoms: ADHD > PTSD > ACEs. More specifically, participants who reported ADHD symptoms obtained higher scores on the Amnesia dimension of the DES-II than PTSD and more severe Absorption subscores than individuals reporting ACEs. Correlational analyses confirmed that DES-II scores were mostly associated with the scale evaluating the severity of ADHD symptoms rather than those evaluating trauma-related manifestations. These findings suggest that neurodevelopmental vulnerabilities, such as ADHD, may play a more significant role in dissociative symptomatology than trauma-related disorders.

## 1. Introduction

The construct of dissociation was first developed by Pierre Janet (Coriat, 1907; Janet, 1907), who defined it as a lack of integration between two or more different “systems of ideas and functions that constitute personality”. Janet (1907) proposed that this phenomenon was caused by an inability to integrate different experiences and develop an awareness of reality as it is, to be able to accept it and then adapt to it in a reflective and creative way. Deficit of integrative capacity might be due to a genetic component, severe illness, fatigue, and particularly adverse and traumatizing experiences (Janet, 1907).

Since the 1980s, several conceptualizations of dissociation have been proposed, both over-inclusive and under-inclusive, often contradictory. Out of these, the “unitary” model proposed that a common psychological mechanism (i.e., dissociation) caused by a deficit in mental integration (Bernstein & Putnam, 1986; Dell, 2006) could be responsible for a wide range of psychological symptoms, states and processes such as absorption, reduced awareness, derealization, and depersonalization. According to this conceptualization, these phenomena are qualitatively similar but differ in the amount of altered state of consciousness as well as in their symptomatology (Steinberg, 2000), giving rise to the concept of dissociative continuum on which the Dissociative Experiences Scale (DES) was developed, a questionnaire used to estimate individual differences in “trait” dissociation (Bernstein & Putnam, 1986).

However, different theoretical formulations support a division between two qualitatively distinct forms of dissociation: compartmentalization and detachment (Allen et al., 1999; Cardeña, 1994; Holmes et al., 2005). Compartmentalization has been defined as a deficit in the ability to deliberately control processes or actions that would typically be under voluntary control, despite these disrupted functions operating normally and continuing to influence cognition, emotion, and action (Holmes et al., 2005). Such deficits cannot be overcome by a simple act of will, but may be reversible, at least in principle (Holmes et al., 2005). In contrast, detachment has been described as an altered state of consciousness characterized by a sense of separation (or “detachment”) from aspects of everyday experiences (Holmes et al., 2005). Phenomenological manifestations of detachment include emotional numbing, out-of-body experiences (a sense of being an outside observer of one’s body), depersonalization, and derealization (alterations in one’s sense of self or the world around) (R. J. Brown, 2006). Although they have different underlying mechanisms, both detachment and compartmentalization may be associated with memory disturbances and amnesia (Allen et al., 1999; Holmes et al., 2005). A more recent global definition proposed by Cardeña and Carlson (2011) described dissociation as “an experienced loss of information or control over mental processes that, under normal circumstances, are available to conscious awareness, self-attribution, or control, depending on the age and cognitive development of the individual”.

Dissociative manifestations may also occur after exposure to traumatic events such as natural disasters, catastrophes, or serious car accidents, and are widely considered core features of trauma-related stress disorders (Carlson et al., 2012; Dalenberg & Carlson, 2012). Much evidence suggests that trauma-related dissociation serves as an automatic protective response that helps individuals detach themselves from intense traumatic experiences (Lanius et al., 2012; Putnam, 1991). Despite the adaptive nature of this response in the short term, its persistence over time might interfere with traumatic memory processing and integration, potentially leading to the onset of post-traumatic stress disorder (PTSD) (Brewin, 2011).

Moreover, the severity of dissociative manifestations has also been linked to adverse childhood experiences (ACEs), including physical maltreatment, sexual abuse, and emotional neglect (Zanarini et al., 1997). For example, in life-threatening and overwhelming situations (e.g., physical aggression and sexual abuse), the body may resort to a passive state ruled by parasympathetic system activation with disruption of the ongoing perceptual and/or bodily experiences such as freezing, emotional numbing, and altered consciousness, hallmarks of shutdown dissociation (Schauer & Elbert, 2010).

Nonetheless, several studies demonstrated the existence of a bidirectional association between ACEs and attention-deficit/hyperactivity disorder (ADHD) symptomatology, suggesting not only that early trauma may increase the risk of developing attention and self-regulation difficulties, but also that children with these traits might also be more prone to ACEs (Lugo-Candelas et al., 2021; Ouyang et al., 2008). In particular, recent studies have revealed that toxic stress due to prolonged or repetitive exposure to ACEs may impact brain development, including regions implicated in ADHD symptomatology, increasing the likelihood of developing the disorder (N. M. Brown et al., 2017; Hughes et al., 2017). On the other hand, ADHD children and, thus, by definition, more impulsive, less self-regulated, and more inattentive, would also be more likely to suffer abuse and maltreatment (Ouyang et al., 2008). In addition, some PTSD manifestations, such as inattention, impulsivity, difficulty concentrating, and restlessness, partially overlap with those of ADHD (Ford & Connor, 2009; Ünver & Karakaya, 2019), which is frequently diagnosed among adolescents in co-occurrence with dissociative disorder (Sar & Ross, 2006).

Recent literature has begun to explore the potential overlap between dissociative manifestations and ADHD-related symptoms. On the one hand, individuals with ADHD may experience alterations in consciousness, such as absorption, spaciness, daydreaming, imaginative involvement, altered time sense, and trance-like behavior (Mette, 2023; Theodor-Katz et al., 2022), which can closely resemble dissociative processes. At the same time, depersonalization and derealization, attention, and consciousness disruptions mimic the manifestations of the inattentive subtype of ADHD, leading to possible misdiagnosis (Foote et al., 2006; Simeon et al., 2003).

These shared features highlight the need to carefully disentangle the trauma-related and neurodevelopmental origins of dissociative manifestations. Identifying whether dissociative symptoms are mostly associated with biological conditions, such as ADHD, or environmental factors, such as trauma-related disorders, might be crucial for treatment purposes in clinical settings. Therefore, to address these purposes, we designed a pilot study from the general population to investigate possible differences in dissociative manifestations associated with acute/event-based trauma, such as PTSD, or chronic/childhood-based trauma due to the occurrence of multiple ACEs, and clinically significant ADHD manifestations.

## 2. Materials and Methods

### 2.1. Participants

We employed a cross-sectional survey methodology to recruit participants through an online survey designed using Google Forms platform. The survey was conducted online for 6 months, and more specifically, data were collected from 1 November 2023, to 30 June 2024.

The survey took approximately 15 min to complete and was disseminated using a snowball sampling strategy to friends, acquaintances, colleagues, and university students, and was also shared on social media platforms (i.e., Facebook, WhatsApp, Instagram, and social virtual groups) to recruit the largest possible sample. Respondents must be at least 18 years old, considering the Italian legislation on adult legal age and compulsory education.

All participants provided their informed consent before completing the online questionnaire. The present study was performed in accordance with the principles that guide the ethical and methodological practice of online research (Das et al., 2011; Hunter et al., 2018) and was approved by the Local Ethics Committee (University of Campania “Luigi Vanvitelli”) and conducted in accordance with the ethical standards laid down in the 1964 Declaration of Helsinki.

### 2.2. Structure of the Survey

The online survey included the following sections:

(i) Informed consent form: participants should provide their consent to be included in the study.
(ii) Sociodemographic data: sex, age, years of formal education (based on the Italian schooling system), marital and employment status, history of neurological and psychiatric conditions or previous psychopathological diagnosis, use of psychotropic drugs or any ongoing psychological treatments, and average hours of sleep per night.
(iii) The Dissociative Experiences Scale-II (DES-II) is a 28-item self-report scale that measures dissociative experiences in daily life related to depersonalization, derealization, amnesia, and absorption (Saggino et al., 2020). This scale is based on the commonly held concept that dissociation is a continuum and thus, each item varies between 0% (“Never”) and 100% (“Always”), describing how often a person has a particular experience. The total DES-II score is represented by the mean of all 28 item scores, with higher total scores associated with dissociative tendencies. According to the three-factor model proposed by Carlson et al. (Carlson et al., 1993), three dimensions can be distinguished: Amnesia, Depersonalization/Derealization and Absorption. Amnesia dimension reflects dissociative memory loss, where individuals experience gaps in their recollection of personal experiences, such as not remembering how they arrived at a place or forgetting actions they have taken. On the other hand, Depersonalization/Derealization dimension encompasses a sense of detachment from oneself and mental processes or a sense of unreality of the self. Individuals may feel as though they are observing their own actions from the outside or experience the world around them as unreal or distorted. Finally, Absorption dimension involves deep immersion in thoughts or experiences, where individuals may become absorbed in mental activities and lose awareness of their surroundings. In addition to the DES-II scores, we computed the DES-taxon score (DES-T), which consists of the average of eight specific DES-II items (i.e., items 3, 5, 7, 8, 12, 13, 22, and 27) and allows for the measurement of pathological dissociation (Waller et al., 1996).
(iv) The Italian version of the Adult ADHD Self-Report Scale (ASRS): patients are asked to rate the frequency of symptoms associated with ADHD they have experienced within the past 6 months. The scale consists of 18 items answered on a 5-point Likert scale from “Never” to “Very often” evaluating both Inattention and Hyperactivity-Impulsivity. Ratings of sometimes, often, or very often on items 1–3, 9, 12, 16, and 18 are assigned one point (ratings of never or rarely are assigned zero points). For the remaining 11 items, ratings of often or very often are assigned one point (ratings of never, rarely, or sometimes are assigned zero points). The symptoms profile of the subjects can be obtained by summing the number of points within each symptom subtype, such that subtype scores can range from 0 to 9. A score ≥ 6 on the Inattention and/or Hyperactivity-Impulsivity subscale is considered symptomatic of ADHD (Adler et al., 2019).
(v) The Impact of Event Scale-Revised (IES-R) was used to evaluate current subjective distress in response to a specific traumatic event (Craparo et al., 2013). The scale consists of 22-items answered on a 5-point Likert scale from 0 (“Not at all”) to 4 (“Extremely”) describing manifestations that may occur after a stressful life event. It comprises three subscales reflecting the major symptom clusters of post-traumatic stress: intrusion, avoidance, and hyper-arousal (Craparo et al., 2013). A score of ≥ 33 indicates a probable diagnosis of PTSD (Creamer et al., 2003).
(vi) The Adverse Childhood Experience questionnaire (ACE) to evaluates adverse childhood experiences. Participants are asked to respond with “Yes” or “No” to 10-items evaluating two sets of adverse childhood experiences: childhood abuse in terms of events that directly affected the respondent (i.e., physical, emotional, or sexual abuse and physical or emotional neglect), and household dysfunction represented by events that happened to other family members and may have indirectly affected the respondent (i.e., parental divorce, domestic violence towards the (step)mother, parental mental health problems, substance use, or imprisonment of a family member) (Van der Feltz-Cornelis & de Beurs, 2023).

### 2.3. Groups Classification

Participants were subdivided into different subgroups based on the presence of ADHD, PTSD, or ACEs symptoms in the following way: individuals who reported scores ≥ 6 on the Inattention and/or Hyperactivity-Impulsivity subscale (Adler et al., 2019) were classified as ADHD; individuals who obtained scores ≥ 33 on IES (Creamer et al., 2003) were considered to have probable PTSD; participants reporting a score ≥ 4 on the ACE questionnaire were considered to present probable childhood trauma.

### 2.4. Statistical Analyses

Sociodemographic and clinical characteristics of the sample were summarized using descriptive statistics.

Then, a parallel approach was adopted to explore how dissociative symptomatology differs across distinct trauma and ADHD profiles. Aim 1: The entire sample was subdivided based on trauma type (i.e., PTSD, ACEs, both, or neither) to assess how dissociative dimensions differed across distinct trauma profiles. Aim 2: Participants were subdivided based on the occurrence of PTSD and/or ADHD (i.e., PTSD, ADHD, both, or neither) to explore possible differences on dissociative manifestations across acute/event-based trauma and ADHD. Aim 3: Participants were classified based on the occurrence of ACEs and/or ADHD (i.e., ACEs, ADHD, both, or neither) to discriminate dissociative symptoms across chronic/childhood-based trauma and ADHD manifestations. To address each of the abovementioned aims, sociodemographic and psychological aspects reported as continuous variables were compared using the Kruskal–Wallis test. Pairwise comparisons with Bonferroni correction were performed to determine the statistically significant differences. A chi-squared (χ^2^) test was used to assess the differences in the distribution of categorical variables.

Pearson correlational analyses were carried out to explore the association between scales evaluating ADHD manifestations and severity of trauma.

The significance level was set at α = 0.05, and all statistical analyses were performed using SPSS Statistic 26.0.

## 3. Results

### 3.1. Participants Characteristics

A total of 400 participants completed the survey without missing data. The sample was balanced for sex (n = 189, 47.25% men and n = 211, 52.75% women), whereas most of the participants aged 18–30 (n = 203, 50.75%) had a master’s degree (n = 223, 55.75%) and were employed (n = 320, 80%). Among our participants, most of them (n = 313, 78.25%) were unmarried/maiden and did not have any sons/daughters (n = 307, 76.75%). Most of participants did not report a diagnosis of psychiatric disorders (n = 376, 94%), 13 participants (3.25%) reported a history of pathological depression/anxiety, 5 participants (1.25%) had obsessive/compulsive disorder, 4 (1%) received a diagnosis of PTSD, and 2 participants (0.50%) had a diagnosis of personality disorder. As for neurodevelopmental disorders, six participants (1.5%) subjectively reported a diagnosis of learning disorder and 3 (0.75%) a diagnosis of ADHD. Only 16 (4%) of the participants were under treatment with antidepressants/anxiolytics, whereas 61 (15.25%) reported ongoing psychological treatment.

### 3.2. How Dissociative Dimensions Differ Across Distinct Trauma Profiles?

#### 3.2.1. Participants Classification According to PTSD and ACEs Manifestations

As for groups subdivision, 180 participants (45%) did not present significant PTSD or ACEs manifestations and were, then, assigned to control group (HC), 14 (3.5%) reported symptoms related to chronic/childhood-based trauma and were assigned to ACE group, 169 participants (42.25%) complained of severe PTSD symptoms but no adverse childhood experiences and were classified as PTSD group, whereas 37 participants (9.25%) reported both PTSD and ACEs symptoms and were assigned to the complex traumatic stress disorder (CTSD) group.

#### 3.2.2. Comparison Between PTSD and ACE Groups on Demographical and Psychological Variables

A significant difference emerged between these groups on age: participants belonging to PTSD group were younger compared to HC group (Table 1). No significant differences between groups emerged considering educational level and sex distribution (Table 1).

As for psychological variables, the three groups differed on ACE and IES scores. More specifically, CTSD and ACE groups scored higher on ACE compared to HC and PTSD groups, but the PTSD group also reported more severe chronic/childhood-based trauma than HC group (Table 1). At the same time, the CTSD and PTSD groups reported higher scores on IES compared to HC and ACE groups (Table 1). Otherwise, considering ADHD manifestations, groups differed on ASRS scores: CTSD and PTSD groups reported more severe ADHD symptoms (ASRS total score) compared to HC group (Table 1). Furthermore, CTSD and PTSD groups obtained higher scores than HC group also when considering Inattention and Hyperactivity-Impulsivity dimensions separately (Table 1).

**Figure 1:**
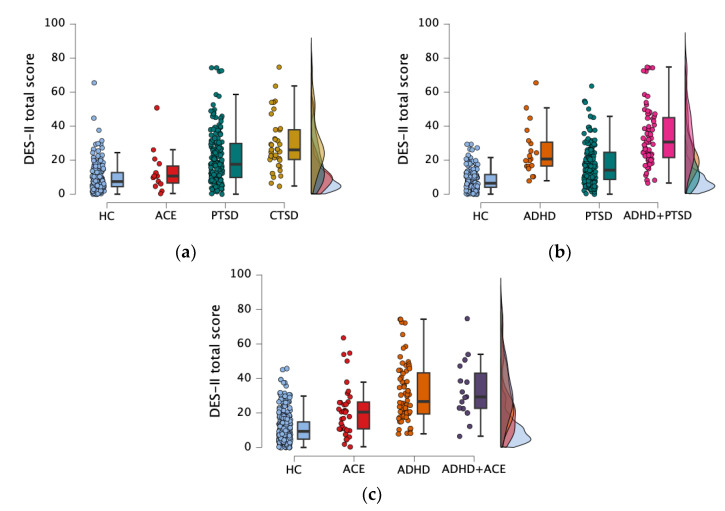
Raincloud plot representing differences in DES-II total score between (**a**) HC, ACE, PTSD, and CTSD groups; (**b**) HC, ADHD, PTSD, and ADHD+PTSD groups; and (**c**) HC, ACE, ADHD, and ADHD+ACE groups. Clouds represent distribution, raindrops represent individual participants, and bars represent 95% confidence intervals.

As for dissociative symptoms, the four groups differed significantly on DES-II subscales and total scores (Table 1). Particularly, CTSD reported higher DES-II scores compared to HC and PTSD or ACE alone, and the PTSD group complained more severe dissociative symptoms than HC group (Figure 1a). As for DES-T, CTSD reported higher scores compared to HC and PTSD alone, whereas the PTSD group reported higher scores compared to HC group (Table 1). Moreover, CTSD group scored higher on DES-II Amnesia and Depersonalization/Derealization subscales than PTSD and HC groups, whereas PTSD reported higher scores on these subscales compared to HC (Table 1). Interestingly, for the Absorption subscale of DES, CTSD and PTSD groups obtained higher scores compared to HC and ACE groups (Table 1).

### 3.3. How Dissociative Dimensions Differ Across PTSD and ADHD Manifestations?

#### 3.3.1. Participants Classification According to PTSD and ADHD Manifestations

As for groups subdivision, 175 participants (43.75%) did not report significant PTSD or ADHD symptoms and constituted the HC group, whereas 19 (4.75%), reported significant ADHD manifestations in the absence of PTSD symptoms and were assigned to the ADHD group. A total of 142 participants (35.5%) complained of severe PTSD symptoms in the absence of ADHD-related manifestations and were classified as the PTSD group, whereas 64 participants (16%) reported both PTSD and ADHD symptoms and were assigned to the ADHD+PTSD group.

#### 3.3.2. Comparison Between PTSD and ADHD Groups on Demographical and Psychological Variables

We found a significant difference on age between the groups: HC were older than individuals in the other groups, and participants belonging to the ADHD+PTSD group were younger compared to those in the PTSD group (Table 2). Moreover, the four groups differed on sex distribution: we observed more males than expected in the HC group (n = 96, 54.86%) than in the ADHD+PTSD group (n = 22, 34.38%), whereas more females than expected were observed in the ADHD+PTSD group (n = 44, 65.63%) than in the HC group (n = 79, 45.14%) (Table 2). No significant difference was observed between the groups in terms of educational level (Table 2).

Moreover, as for psychological symptoms, the PTSD and ADHD+PTSD groups reported higher scores on IES than the HC and ADHD groups, whereas the ADHD and ADHD+PTSD groups complained of more severe ADHD manifestations (ASRS total and Inattention and Hyperactivity-Impulsivity subscores) than PTSD and HC groups (Table 2). At the same time, the four groups differed on chronic/childhood-based trauma: ADHD+PTSD scored higher on ACE compared to PTSD alone and HC groups, whereas the PTSD group reported higher scores than HC group (Table 2).

As for dissociative symptoms, HC scored lower on DES-II total score and DES-T than the ADHD, PTSD, and ADHD+PTSD groups, and the PTSD group reported lower scores than the ADHD+PTSD group (Table 2; Figure 1b).

Interestingly, the ADHD, PTSD, and ADHD+PTSD groups scored higher on the DES-II Amnesia subscale than HC group whereas the ADHD and ADHD+PTSD reported higher scores on this subscale than the PTSD group (Table 2). Moreover, for Depersonalization/Derealization and Absorption subscales of the DES, the ADHD, PTSD, and ADHD+PTSD groups reported higher scores than the HC group, and ADHD+PTSD scored higher than the PTSD alone group (Table 2).

### 3.4. How Dissociative Dimensions Differ Across ACEs-Related and ADHD Manifestations?

#### 3.4.1. Participants Classification According to ACEs-Related and ADHD Manifestations

Participant classification showed that 281 participants (70.25%) did not report significant ADHD manifestations or ACE-related symptoms and constituted, and then the HC group. Conversely, 36 (9%) reported a history of ACEs in the absence of ADHD manifestations and were assigned to the ACE group, 68 participants (17%) complained of severe ADHD symptoms without a history of ACEs and were classified as the ADHD group, whereas 15 participants (3.75%) reported both ACEs and ADHD symptoms and were assigned to the ADHD+ACE group.

#### 3.4.2. Comparison Between ACE and ADHD Groups on Demographical and Psychological Variables

The groups differed on age and years of schooling: ADHD individuals were younger than ACE and HC groups, whereas the ACE group had a lower educational level than the HC group (Table 3). However, no significant difference emerged between the groups in terms of sex distribution (Table 3).

Moreover, as for psychological symptoms, the ADHD and ADHD+ACE groups reported higher total scores and Inattention subscores of ASRS than the HC and ACE groups, with ACE reporting more severe ADHD manifestation than the HC group. Conversely, as for ASRS Hyperactivity-Impulsivity subscore, ADHD and ADHD+ACE groups reported higher scores than HC and ACE alone groups (Table 3). At the same time, the four groups differed on ACE scores: the ADHD+ACE and ACE scored higher on ACE compared to ADHD and HC groups, whereas ADHD group reported more adverse childhood experiences than HC group (Table 3). Conversely, the ADHD+ACE, ADHD, and ACE groups reported more severe acute/event-based trauma (using the IES) than the HC group (Table 3).

As for dissociation, the ADHD+ACE, ADHD, and ACE groups reported higher scores on DES-T, DES-II total score, and all its subscales compared to HC group, whereas ADHD showed higher total (Figure 1c) and Absorption scores compared to ACE (Table 3).

### 3.5. Correlation Analyses

**Figure 2:**
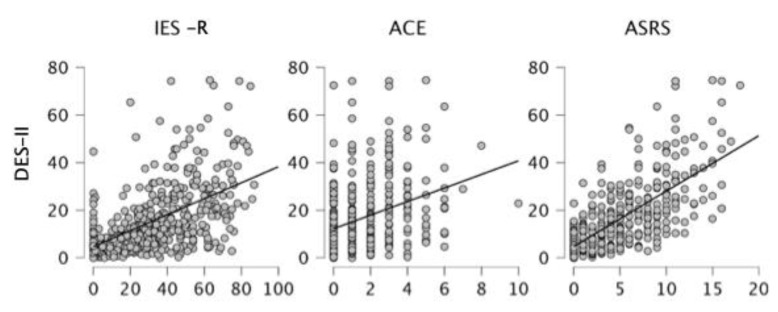
Scatterplot showing the correlation between DES-II total score and three other variables: IES-R, ACE, and ASRS. Each point represents an individual participant, with trend lines indicating the direction and strength of the associations.

Correlational analyses between variables exploring dissociation, ADHD, and trauma revealed that DES-II total score was associated with ASRS total score (r = 0.691, *p* < 0.001), Inattention (r = 0.622, *p* < 0.001), Hyperactivity-Impulsivity (r = 0.623, *p* < 0.001) subscales, IES (r = 0.517, *p* < 0.001), and ACE (r = 0.339, *p* < 0.001) (Figure 2). The same pattern of associations was found also when considering DES-T: moderate to strong associations with ASRS scores (total score: r = 0.618, *p* < 0.001; Inattention: r = 0.542, *p* < 0.001; Hyperactivity-Impulsivity: r = 0.575, *p* < 0.001), moderate association with IES (r = 0.485, *p* < 0.001), and weaker with ACE (r = 0.342, *p* < 0.001). As for DES-II subscales, ADHD manifestations severity was more associated with Absorption subscale of DES-II (r = 0.673, *p* < 0.001) rather than Amnesia (r = 0.556, *p* < 0.001) and Depersonalization/Derealization (r = 0.518, *p* < 0.001) with Inattention and Hyperactivity-Impulsivity dimensions of ADHD following the same pattern of associations. Also, acute/event-related trauma (assessed by IES) was more strongly related to Absorption dimension of DES-II (r = 0.504, *p* < 0.001) rather than Depersonalization/Derealization (r = 0.421, *p* < 0.001) and Amnesia (r = 0.381, *p* < 0.001). Conversely, chronic/childhood-related trauma (assessed by ACE) was more associated with Depersonalization/Derealization dimension of DES-II (r = 0.311, *p* < 0.001) respect to Absorption (r = 0.266, *p* < 0.001) and Amnesia (r = 0.297, *p* < 0.001). 

## 4. Discussion

We aimed to explore the complex interplay between different types of trauma, ADHD symptoms, and dissociative manifestations. To achieve this, in this pilot study, we adopted a parallel approach on a large sample from the general population by separately analyzing pairwise comparisons. First, we analyzed how dissociative dimensions differed across different trauma types (PTSD and/or ACE-related manifestations). Second, we explored patterns of dissociation based on the occurrence of ADHD and/or PTSD manifestations. Third, we explored differences on dissociative phenomena between participants who reported ADHD and/or ACE-related symptoms.

We found that dissociative symptoms were more pronounced in participants with combined ADHD and PTSD or ACEs manifestations. However, significant variations in dissociative experiences also emerged across isolated symptoms of different types of trauma exposure and ADHD occurrence, with dissociation severity following this hierarchical pattern: ADHD > PTSD > ACEs. These findings were substantially confirmed when considering the DES-T, which quantifies the pathological dissociation.

More specifically, individuals with PTSD-related symptoms reported higher scores on the Absorption dimension than those with ACEs; furthermore, individuals with only ADHD-like manifestations showed higher scores on the Amnesia susbscale than individuals with PTSD and higher Absorption scores than individuals with ACEs alone. Correlation analysis confirmed that dissociation was more strongly associated with ADHD severity and PTSD than with ACEs. Thus, the Absorption dimension was associated with both ADHD and PTSD severity, while the Amnesia subscale was more strongly related to ADHD manifestations than PTSD severity. Conversely, ACEs were more strongly related to the Depersonalization/Derealization subscale than to the other dissociation dimensions.

Overall, our findings further support the observation that dissociative symptoms can occur across a range of psychiatric disorders, representing a potential confounding factor in both diagnostic assessment and treatment planning (Sar & Ross, 2006). However, potential fluctuations in symptom severity, along with differences across various dimensions of dissociation, may help clinicians distinguish between dissociative features associated with genetic and neurodevelopmental conditions and those resulting from traumatic experiences.

We showed that individuals presenting with both post-traumatic manifestations and ADHD symptomatology reported more severe dissociation than those presenting with traumatic experiences alone. Moreover, individuals with only ADHD traits reported higher DES-II scores than those with PTSD or ACEs alone. These findings suggest that neurobiological vulnerability, particularly when linked to a neurodevelopmental condition, appears to play a more significant role in the development of dissociative disorders compared to environmental factors, such as traumatic experiences. Severe dissociative manifestations in ADHD can be explained as a consequence of disrupted brain mechanisms due to neurodevelopmental factors (Arnsten, 2009; Bob & Konicarova, 2018; Makris et al., 2009). Such neural disorganization may disrupt the integration of mental processes, affecting attention, memory, and consciousness (core components of psychological awareness), thereby increasing vulnerability to dissociative phenomena, where the continuity of consciousness and awareness is fragmented (Bob & Konicarova, 2018). However, it should be emphasized that it is difficult to distinguish ADHD from dissociative manifestations, since the latter may mimic typical ADHD features such as absorption and inattention (Endo et al., 2006).

More specifically, we found that individuals with ADHD reported higher scores on the Amnesia subscale of the DES-II than those with PTSD. Our results are further supported by Giesbrecht et al. (2004), who found that dissociative amnesia was related to minor disruptions in executive functioning in a sample of young adults. Indeed, ADHD manifestations include difficulties in sustained attention (Fuermaier et al., 2022) and working memory (Matt Alderson et al., 2013), which can generate “gaps” in awareness, where individuals may not fully encode or consolidate events or experiences into their memory. Hence, people with ADHD may be distracted while completing routine tasks such as performing an activity or moving from a place to another and, therefore, may fail to form a coherent memory and recall specific details about how they got somewhere or performed a task (e.g., shopping or getting dressed), experiences described in the Amnesia factor of the DES-II (Carlson et al., 1993). In contrast, PTSD has also been associated with memory impairments, such as memory fragmentation due to inadequate encoding (Bedard-Gilligan & Zoellner, 2012) and partial amnesia as a defense mechanism that protects the individual by avoiding the traumatic experience (Berntsen & Rubin, 2014; Clark & Ehlers, 2000; van der Kolk & Fisler, 1995).

Nevertheless, hypervigilance represents a key hyperarousal symptom of PTSD, characterized by an enhanced state of sensory sensitivity accompanied by an exaggerated intensity of behaviors to be constantly on guard and scan for potential threats (Adams et al., 2020; Eisenberg et al., 2023). However, this symptom can manifest as restlessness, irritability, and heightened startle responses, which are behaviors that may superficially resemble the hyperactivity and impulsivity observed in ADHD (Adams et al., 2020).

These similar manifestations can complicate the differential diagnosis between PTSD and ADHD, particularly in individuals with a history of trauma. Neurobiological studies have delineated distinct patterns associated with these conditions. For instance, the hyperaroused subtype of PTSD is linked to emotional undermodulation due to insufficient prefrontal inhibition of limbic regions, whereas ADHD is associated with deficits in executive functioning and attention regulation. Recognizing these nuanced differences is crucial for accurate diagnosis and effective treatment planning.

In addition, scores on the Absorption subscale emerged as a distinguishing factor between individuals reporting ACEs and those with ADHD, with the latter reporting a more intense state of absorption that disrupts their awareness of the surrounding environment. The way in which ADHD affects attention, imagination, and engagement with external and internal stimuli may explain the high scores on the Absorption subscale, as it includes items related to being unaware of the passage of time or difficulty distinguishing between daydreaming and reality (Carlson et al., 1993). Indeed, individuals with ADHD often slip into daydreaming, immersing themselves in extremely vivid fantasies or past experiences, making it challenging for them to return to reality or distinguish their daydreams from real events (Somer et al., 2017; Theodor-Katz et al., 2022). Additionally, ADHD-related hyperfocus is characterized by intense concentration on pleasurable or rewarding activities or tasks, making it difficult to shift attention, leading to reduced awareness of the self, the external environment, and the passage of time (Groen et al., 2020). These findings further reflect the diagnostic challenges posed by the symptomatic overlap between ADHD and dissociation, particularly in trauma-exposed individuals.

Interestingly, participants reporting ACEs showed less severe dissociation than those with PTSD and ADHD, particularly in relation to the Absorption dimension. Indeed, ACEs lead to more general symptoms such as affective dysregulation, negative self-concept, and interpersonal issues (Cloitre et al., 2013) along with typical post-traumatic disorder and show a higher prevalence of dissociation compared to PTSD (Dalenberg & Carlson, 2012; Longo et al., 2019). Hence, our results appear to be in contrast with those of previous studies demonstrating more severe dissociative manifestations in complex trauma than in acute PTSD (Cloitre et al., 2013; Dalenberg & Carlson, 2012; Hyland et al., 2018; Longo et al., 2019), which may be due to the different methodological approaches. In the present study, we collected data about traits and manifestations related to trauma, ADHD, and dissociation within the general population, which typically presents less pronounced and less consistent symptomatology than clinical populations with formal diagnoses, and this might affect the prevalence, severity, and pattern of symptoms observed. More specifically, most participants classified as having ACEs (n = 51) most frequently complained of emotional neglect (e.g., feeling unloved, 78.4%) and emotional abuse (e.g., being denigrated and insulted, 94.1%), whereas reports of physical or sexual abuse as well as those of household dysfunction (including history of substance abuse, incarceration, and domestic violence) were less frequent with prevalence rates ranging from 17.6% to 54.9%. Considering that the type and timing of ACEs influence psychological outcomes through unique pathways (Schalinski et al., 2016), our participants reported primarily emotional neglect rather than other forms of ACEs might limit the comparability of our results with those of previous studies.

However, it is possible to speculate that individuals presenting ACEs, although experiencing broader symptomatology, tend to use avoidance and emotional numbing as coping mechanisms to distance themselves from distressing memories (Cloitre et al., 2013) rather than fully immersing themselves in traumatic memories or daydreaming as in PTSD, thus obtaining lower absorption scores.

At the same time, experiences of derealization and depersonalization may act as protective mechanisms that shield individuals from emotional pain and trauma (Dalenberg & Carlson, 2012), which may also explain the close relationship between ACEs and Depersonalization/Derealization DES-II subscale compared to other dimensions. Indeed, ACEs such as abuse and neglect promote this shutdown dissociation (derealization and depersonalization) as a coping mechanism to withstand overwhelming trauma (Schalinski et al., 2015), making them more prominent in ACEs than transient phenomena like dissociative amnesia (typically occurring in acute trauma survivors (Van der Hart & Brom, 2000; Yehuda et al., 2014)) or absorption (a general psychological trait rather than a specific trauma manifestation (Lyssenko et al., 2018)) that do not directly mitigate emotional overwhelm.

However, it should be emphasized that there are some limitations to the generalizability of these results.

First, our participants were recruited by a snowball strategy from the general population, and this, together with the cross-sectional nature of the study that does not allow for longitudinal and/or causal inferences, could limit the generalization of the results despite the large sample size. However, these choices were motivated by the exploratory nature of the study and the need to recruit a large sample within a limited timeframe.

Moreover, we employed screening questionnaires such as the ASRS, ACE, and IES to evaluate the severity and/or frequency of ADHD and trauma-related manifestations without providing definitive evidence for specific diagnostic labels. To this, our group subdivision reflects a symptom-based classification rather than a clinical diagnosis, consistent with the approaches adopted in other population-based studies.

Furthermore, our findings from the comparison analyses were based on heterogeneous group sizes. For example, the existing bidirectional association between ADHD symptomatology and ACEs (Lugo-Candelas et al., 2021; Ouyang et al., 2008), with the occurrence of one increasing the likelihood of the co-occurrence of the other (Lugo-Candelas et al., 2021; Ouyang et al., 2008), may explain the limited number of individuals with “pure” ADHD or ACEs that could affect the results of the comparison analyses and should, therefore, be interpreted with caution.

In addition, it should be noted that we exclusively recruited an adult sample, which did not allow us to explore the ADHD/trauma-dissociation interaction at earlier stages of life. Indeed, considering that adult presentation of ADHD diverges from its manifestation during childhood with an age-related decline of symptoms (Faraone et al., 2006), further studies on childhood and adolescence may help in understanding the phenomenon at its full expression.

Overall, it should be considered that the present work may serve as a pilot study to provide, for the first time, preliminary insights into the complex interplay between ADHD symptoms, trauma exposure, and dissociative manifestations.

Therefore, future studies should aim to address the abovementioned aspects by enrolling clinically diagnosed participants to confirm our preliminary observations and including children and adolescents to capture the early manifestations of these phenomena. In addition, longitudinal studies may reveal the developmental trajectories of symptoms and their interrelations over time.

## 5. Conclusions

In conclusion, our findings provide preliminary evidence of the association between dissociative phenomena and trauma-related and ADHD manifestations. We revealed that dissociation may arise from both neurodevelopmental vulnerabilities and traumatic experiences, with participants with relevant ADHD symptoms demonstrating more severe dissociative symptoms than those with trauma-related manifestations. Furthermore, significant differences in dissociative dimensions, such as the prominence of Amnesia in participants with more severe ADHD manifestations, may guide clinicians distinguish between dissociative symptoms associated with neurodevelopmental conditions or trauma exposure in order to implement tailored interventions. The present results further confirm the clinical complexity of neurodevelopmental and trauma-related symptomatology and emphasize the need for comprehensive assessments that consider developmental history, trauma exposure, and the context of symptom emergence to disentangle these presentations effectively.

## Figures and Tables

**Table 1:** Comparison of groups on sociodemographic and neuropsychiatric variables across different traumatic conditions.

	HC Group (n = 180) A	ACE Group (n = 14) B	PTSD Group (n = 169) C	CTSD Group (n = 37) D		
	Mean ± SD	Mean ± SD	Mean ± SD	Mean ± SD	χ^2^	*p*
Age	34.30 ± 10.57	34.29 ± 10.34	29.83 ± 7.16	31.14 ± 11.78	21.779	**<0.001** **C < A**
Educational level (ys)	16.14 ± 2.74	13.71 ± 4.43	15.80 ± 2.59	15.00 ± 3.11	7.117	0.068
Sex	97 M/83 F	6 M/8 F	67 M/102 F	19 M/18 F	7.463	0.059
**Neuropsychiatric Variables**						
IES-R	15.35 ± 11.05	22.64 ± 6.52	51.63 ± 13.79	58.11 ± 13.73	302.281	**<0.001** **C > A,B** **D > A,B**
ACE questionnaire	0.76 ± 0.96	4.64 ± 0.84	1.17 ± 1.13	5.08 ± 1.32	153.828	**<0.001** **A < B,C,D** **C < B,D**
DES-II total	9.62 ± 8.67	13.57 ± 12.81	21.04 ± 15.50	29.36 ± 16.13	100.085	**<0.001** **D > A,B,C** **C > A**
DES-T	6.64 ± 7.96	13.12 ± 12.32	16.74 ± 15.00	25.27 ± 16.85	93.278	**<0.001** **D > A,C** **C > A**
DES-II Amnesia	4.97 ± 6.85	10.36 ± 11.53	11.93 ± 13.77	18.96 ± 14.02	63.073	**<0.001** **A < C,D** **D > C**
DES-II Depersonalization	2.87 ± 7.35	9.28 ± 17.67	11.91 ± 16.77	20.40 ± 21.66	73.734	**<0.001** **A < C,D** **D > C**
DES-II Absorption	16.81 ± 14.71	16.55 ± 16.35	33.56 ± 22.56	40.81 ± 19.20	87.316	**<0.001** **A < C,D** **B < C,D**
ASRS Impulsiveness	1.31 ± 1.60	1.93 ± 1.54	2.95 ± 2.32	3.62 ± 2.09	69.214	**<0.001** **A < C,D**
ASRS Inattention	1.89 ± 2.32	2.86 ± 2.35	3.37 ± 2.77	4.22 ± 2.17	44.173	**<0.001** **A < C,D**
ASRS total	3.20 ± 3.37	4.79 ± 3.62	6.31 ± 4.55	7.84 ± 3.77	66.217	**<0.001** **A < C,D**

HC = Healthy controls; ACE = Adverse Childhood Experience; PTSD = Post-traumatic stress disorder; CTSD = Complex traumatic stress disorder; IES-R = Impact of Event Scale-Revised; DES-II = Dissociative Experiences Scale-II; DES-T = DES-taxon; ASRS = Adult attention-deficit/hyperactivity disorder Self-Report Scale.

**Table 2:** Comparison of groups on sociodemographic and neuropsychiatric variables between PTSD and ADHD groups.

	HC (n = 175) A	ADHD (n = 19) B	PTSD (n = 142) C	ADHD+PTSD (n = 64) D		
	Mean ± SD	Mean ± SD	Mean ± SD	Mean ± SD	χ^2^	*p*
Age	34.86 ± 10.76	29.11 ± 6.14	30.85 ± 8.38	28.33 ± 7.44	38.587	**<0.001** **A > B,C,D** **D < C**
Educational level (ys)	15.89 ± 2.99	16.68 ± 2.43	15.81 ± 2.50	15.33 ± 3.10	4.470	0.215
Sex	96 M/79 F	7 M/12 F	64 M/78 F	22 M/42 F	9.416	**0.024**
**Neuropsychiatric Variables**						
IES-R	15.97 ± 10.79	15.00 ± 12.53	49.63 ± 12.00	59.81 ± 15.51	304.338	**<0.001** **A < C,D** **B < C,D**
ACE questionnaire	1.03 ± 1.41	1.11 ± 1.20	1.64 ± 1.81	2.38 ± 2.00	31.929	**<0.001** **A < C,D** **C < D**
DES-II total	8.21 ± 6.19	25.51 ± 14.92	17.34 ± 12.15	34.06 ± 17.21	157.060	**<0.001** **A < B,C,D** **C < D**
DES-T	6.07 ± 6.16	16.71 ± 17.17	13.61 ± 11.37	28.61 ± 18.71	115.857	**<0.001** **A < B,C,D** **C < D**
DES-II Amnesia	4.14 ± 5.45	16.58 ± 12.21	9.86 ± 10.52	20.60 ± 17.70	95.462	**<0.001** **A < B,C,D** **C < B, D**
DES-II Depersonalization	2.02 ± 4.11	15.44 ± 21.37	9.17 ± 13.23	22.92 ± 22.98	95.927	**<0.001** **A < B,C,D** **C < D**
DES-II Absorption	14.23 ± 11.44	40.44 ± 20.60	27.63 ± 18.51	50.91 ± 21.14	148.789	**<0.001** **A < B,C,D** **C < D**
ASRS Impulsiveness	1.12 ± 1.28	3.53 ± 2.50	2.20 ± 1.73	4.98 ± 2.24	129.224	**<0.001** **A < B,C,D** **C < D**
ASRS Inattention	1.43 ± 1.54	6.84 ± 1.80	2.11 ± 1.72	6.66 ± 1.58	190.072	**<0.001** **A<B,C,D** **C < B,D**
ASRS total	2.55 ± 2.41	10.37 ± 3.15	4.31 ± 3.01	11.64 ± 2.55	202.215	**<0.001** **A < B,C,D** **C < B,D**

HC = Healthy controls; ACE = Adverse Childhood Experience; PTSD = Post-traumatic stress disorder; ADHD = Attention-deficit/hyperactivity disorder; ADHD+PTSD = Combined ADHD and PTSD manifestations; IES-R = Impact of Event Scale-Revised; DES-II = Dissociative Experiences Scale-II; DES-T = DES-taxon; ASRS = Adult attention-deficit/hyperactivity disorder Self-Report Scale.

**Table 3:** Comparison of groups on sociodemographic and neuropsychiatric variables between ACE and ADHD groups.

	HC (n = 281) A	ADHD (n = 68) B	ACE (n = 36) C	ADHD+ACE (n = 15) D		
	Mean ± SD	Mean ± SD	Mean ± SD	Mean ± SD	χ^2^	*p*
Age	33.05 ± 9.76	28.34 ± 6.06	33.14 ± 11.50	29.27 ± 11.02	25.433	**<0.001** **B < A,C**
Educational level (ys)	16.09 ± 2.60	15.51 ± 2.89	14.00 ± 3.36	16.20 ± 3.51	12.741	**0.005** **C < A**
Sex	141 M/140 F	23 M/45 F	19 M/17 F	6 M/9 F	2.667	0.102
**Neuropsychiatric Variables**						
IES-R	29.41 ± 19.79	47.41 ± 24.77	43.83 ± 19.28	59.27 ± 18.09	55.922	**<0.001** **A < B,C,D**
ACE questionnaire	0.84 ± 1.00	1.43 ± 1.18	4.92 ± 0.87	5.07 ± 1.83	155.960	**<0.001** **A < B,C,D** **B < C,D**
DES-II total	11.13 ± 8.96	31.77 ± 17.00	21.47 ± 15.26	33.57 ± 17.59	123.419	**<0.001** **A < B,C,D** **B > C**
DES-T	8.29 ± 8.33	24.93 ± 18.70	18.47 ± 13.69	30.25 ± 20.11	92.949	**<0.001** **A < B,C,D**
DES-II Amnesia	5.65 ± 7.19	19.46 ± 17.08	14.91 ± 13.21	20.67 ± 14.88	91.960	**<0.001** **A < B,C,D**
DES-II Depersonalization	4.09 ± 7.87	20.32 ± 21.95	14.07 ± 17.84	25.22 ± 26.40	80.037	**<0.001** **A < B,C,D**
DES-II Absorption	19.06 ± 15.35	49.17 ± 22.20	29.40 ± 21.22	45.55 ± 17.34	110.728	**<0.001** **A < B,C,D** **B > C**
ASRS Impulsiveness	1.53 ± 1.60	4.46 ± 2.49	2.17 ± 1.38	5.53 ± 1.46	106.648	**<0.001** **A < B,D** **C < B,D**
ASRS Inattention	1.59 ± 1.61	6.79 ± 1.60	2.83 ± 1.65	6.27 ± 1.71	192.666	**<0.001** **A < B,C,D** **C < B,D**
ASRS total	3.12 ± 2.80	11.25 ± 2.86	5.00 ± 2.55	11.80 ± 2.08	192.106	**<0.001** **A < B,C,D** **C < B,D**

HC = Healthy controls; ACE = Adverse Childhood Experience; ADHD = Attention-deficit/hyperactivity disorder; ADHD+ACE = Combined ADHD and ACE manifestations; IES-R = Impact of Event Scale-Revised; DES-II = Dissociative Experiences Scale-II; DES-T = DES-taxon; ASRS = Adult attention-deficit/hyperactivity disorder Self-Report Scale.

## Data Availability

Data cannot be shared openly to protect study participants’ privacy. However, data and analysis code will be available upon request to corresponding author.

## Abbreviations

The following abbreviations are used in this manuscript:

ACEs	Adverse Childhood Experiences
ADHD	Attention-Deficit/Hyperactivity Disorder
ASRS	Adult ADHD Self-Report Scale
CTSD	Complex traumatic stress disorders
DES	Dissociative Experience Scale
IES-R	Impact of Event-Revised Scale
PTSD	Post-Traumatic Stress Disorder
